# Transient Depletion of Foxp3^+^ Regulatory T Cells Selectively Promotes Aggressive β Cell Autoimmunity in Genetically Susceptible DEREG Mice

**DOI:** 10.3389/fimmu.2021.720133

**Published:** 2021-08-10

**Authors:** Deepika Watts, Marthe Janßen, Mangesh Jaykar, Francesco Palmucci, Marc Weigelt, Cathleen Petzold, Angela Hommel, Tim Sparwasser, Ezio Bonifacio, Karsten Kretschmer

**Affiliations:** ^1^Molecular and Cellular Immunology/Immune Regulation, Center for Regenerative Therapies Dresden (CRTD), Center for Molecular and Cellular Bioengineering (CMCB), Technische Universität Dresden, Dresden, Germany; ^2^Paul Langerhans Institute Dresden (PLID) of the Helmholtz Zentrum München at the University Hospital and Medical Faculty Carl Gustav Carus of TU Dresden, Dresden, Germany; ^3^German Center for Diabetes Research (DZD e.V.), Neuherberg, Germany; ^4^Regenerative Therapies for Diabetes, Center for Regenerative Therapies Dresden (CRTD), Center for Molecular and Cellular Bioengineering (CMCB), Technische Universität Dresden, Dresden, Germany; ^5^Institute of Infection Immunology, TWINCORE/Centre for Experimental and Clinical Infection Research, Hanover, Germany

**Keywords:** type 1 diabetes, immune regulation, Treg cells, Foxp3, cell ablation

## Abstract

Type 1 diabetes (T1D) represents a hallmark of the fatal multiorgan autoimmune syndrome affecting humans with abrogated Foxp3^+^ regulatory T (Treg) cell function due to *Foxp3* gene mutations, but whether the loss of Foxp3^+^ Treg cell activity is indeed sufficient to promote β cell autoimmunity requires further scrutiny. As opposed to human Treg cell deficiency, β cell autoimmunity has not been observed in non-autoimmune-prone mice with constitutive *Foxp3* deficiency or after diphtheria toxin receptor (DTR)-mediated ablation of Foxp3^+^ Treg cells. In the spontaneous nonobese diabetic (NOD) mouse model of T1D, constitutive Foxp3 deficiency did not result in invasive insulitis and hyperglycemia, and previous studies on Foxp3^+^ Treg cell ablation focused on Foxp3^DTR^ NOD mice, in which expression of a transgenic BDC2.5 T cell receptor (TCR) restricted the CD4^+^ TCR repertoire to a single diabetogenic specificity. Here we revisited the effect of acute Foxp3^+^ Treg cell ablation on β cell autoimmunity in NOD mice in the context of a polyclonal TCR repertoire. For this, we took advantage of the well-established DTR/GFP transgene of DEREG mice, which allows for specific ablation of Foxp3^+^ Treg cells without promoting catastrophic autoimmune diseases. We show that the transient loss of Foxp3^+^ Treg cells in prediabetic NOD.DEREG mice is sufficient to precipitate severe insulitis and persistent hyperglycemia within 5 days after DT administration. Importantly, DT-treated NOD.DEREG mice preserved many clinical features of spontaneous diabetes progression in the NOD model, including a prominent role of diabetogenic CD8^+^ T cells in terminal β cell destruction. Despite the severity of destructive β cell autoimmunity, anti-CD3 mAb therapy of DT-treated mice interfered with the progression to overt diabetes, indicating that the novel NOD.DEREG model can be exploited for preclinical studies on T1D under experimental conditions of synchronized, advanced β cell autoimmunity. Overall, our studies highlight the continuous requirement of Foxp3^+^ Treg cell activity for the control of genetically pre-installed autoimmune diabetes.

## Introduction

Type 1 diabetes (T1D) is a chronic disease under complex environmental, immunological and genetic control, which is manifested by the autoimmune destruction of functional insulin-producing β cells of pancreatic islets caused by islet-infiltrating diabetogenic CD4^+^ and CD8^+^ T cells (1, 2). The concept of Foxp3^+^ regulatory T (Treg) cell-based therapies to interfere with β cell autoimmunity in T1D has been fueled in large part by evidence indicating that diminished Treg cell activity may contribute to disease pathogenesis. In fact, many of the T1D genetic susceptibility loci have been implicated in Foxp3^+^ Treg cell function, either through indirect (e.g., *IL2*) or direct (e.g., *IL2R, CTLA4, PTPN22, IL10*) mechanisms (2). Likewise, T1D is considered a hallmark (3) of the fatal multiorgan autoimmune syndrome (IPEX; immune dysfunction, polyendocrinopathy, enteropathy, X-linked) affecting humans with abrogated Treg cell function due to genetic *FOXP3* gene mutations (4, 5), demonstrating that a severe Treg cell defect is sufficient to promote destructive β cell autoimmunity independently of other genetic and environmental factors. However, loss-of-function studies in non-autoimmune and diabetes-prone mice have not been able to demonstrate an unequivocal link between Foxp3^+^ Treg cell deficiency and catastrophic β cell autoimmunity. In mice on a non-autoimmune genetic background, constitutive genetic *Foxp3* deficiency (6, 7) or acute Foxp3^+^ Treg cell ablation based on Foxp3-driven expression of a human diphtheria toxin receptor (DTR) (8–10) recapitulates many clinical features of the human IPEX syndrome, but the manifestation of β cell autoimmunity has not been reported. Moreover, Foxp3-deficient *scurfy* mice on the diabetes-prone NOD background (NOD.Foxp3*^sf^*) develop exocrine pancreatitis and peri-insulitis, but do not develop insulitis and overt diabetes, unless the CD4^+^ T cell receptor (TCR) repertoire of NOD.Foxp3*^sf^* mice is artificially restricted to a single highly diabetogenic specificity by transgenic expression of the BDC2.5 TCR (11). These observations could be interpreted as evidence that Foxp3^+^ Treg cells are dispensable for the autoimmune β cell protection in the NOD model, but the interpretation of data in the constitutive absence of Treg cells is hampered by potentially confounding effects of immune adaptations and severe systemic autoimmunity, including premature death and alterations in thymic T cell development (12). The administration of mAbs directed against CD25 as ‘surrogate’ Treg cell marker largely preserves systemic immune homeostasis and has been employed to examine the role of Foxp3^+^ Treg cells in the control of β cell autoimmunity. However, CD25 is not uniquely expressed on Foxp3^+^ Treg cells, and anti-CD25 mAb treatment has been proposed to act on Treg cells by functional inactivation, rather than physical depletion (13–15), while sparing Foxp3^+^ Treg cells with a CD25^–^ phenotype. These limitations in specificity and efficiency of CD25-targeted interference with Foxp3^+^ Treg cell activity may account for the largely contradictory range of data in the NOD model, including precipitation of overt diabetes (16–19), accelerated diabetes progression in young but not adult mice (20), as well as maintenance of β cell tolerance (21–23), or even delayed onset of diabetes (22).

Foxp3-driven DTR expression has been successfully employed for the specific and temporally controlled ablation of Foxp3^+^ Treg cells (8–10, 24), while the outcome can considerably differ between independent Foxp3^DTR^ mouse lines, depending on the transgenic strategy of Foxp3-driven DTR/GFP expression. In ‘knock-in’ mice expressing a DTR/GFP fusion protein from an IRES down-stream of the endogenous *Foxp3* gene (Foxp3^IRES-DTR/GFP^) on a non-autoimmune genetic background, DT-mediated Treg cell-ablation in young and adult Foxp3^IRES-DTR/GFP^ mice resulted in an autoimmune disease similar to that observed in Foxp3-deficient mice (8). In the DEREG (‘depletion of regulatory T cell’) model, in which DTR/GFP is expressed from a transgenic Foxp3 bacterial artificial chromosome (Foxp3^BAC-DTR/GFP^), administration of DT into newborns resulted in *scurfy*-like symptoms, while adults were found to be protected from autoimmune diseases, despite efficient GFP^+^ Treg cell depletion (9). In fact, and as opposed to Foxp3^IRES-DTR/GFP^ mice, the Foxp3^BAC-DTR/GFP^ transgene of DEREG mice is not physically linked to the endogenous *Foxp3* gene, allowing for the accumulation of DT-resistant Foxp3^+^GFP^–^ Treg cells (25–28). These unique features prompted us to hypothesize that the DEREG model is particularly suited to study the role of Foxp3^+^ Treg cells in organ-specific autoimmunity in mice on a genetically susceptible genetic background, while keeping collateral autoimmune damage to a minimum. In previous studies employing a NOD model, in which the spontaneous diabetes development was constrained by transgenic BDC2.5 TCR expression on all CD4^+^ T cells (29), acute Foxp3^+^ Treg cell ablation was shown to unleash a highly aggressive form of autoimmune diabetes (30), which fully abrogated the sex bias usually observed in spontaneous diabetes progression of NOD mice (31). Here, we report on the specific effects of acute Foxp3^+^ Treg cell ablation on the physiologic disease course in autoimmune diabetes-prone DEREG mice in the context of a polyclonal TCR repertoire.

## Materials and Methods

### Mice

NOD/ShiLtJ, NOD.Rag1^–/–^, NOD.BDC2.5 mice (all Jackson Laboratories, Bar Harbor, USA), and DEREG mice (9) on different genetic backgrounds (C57BL/6, BALB/c, or NOD) were housed and bred at the Animal Facility of the CRTD under specific pathogen-free conditions. NOD.DEREG mice were obtained by backcrossing C57BL/6.DEREG mice onto the NOD/ShiLtJ background for ≥13 generations. NOD.DEREG mice were intercrossed with NOD.BDC2.5 mice to obtain NOD.DEREG × BDC2.5 mice. All NOD mouse lines were fed with NIH #31M rodent diet (Altromin, Germany). Blood glucose levels were measured using whole blood from the tail vein and Accu-Chek^®^ Aviva (Roche). If not stated otherwise, blood glucose levels were routinely determined once a week. Mice were considered diabetic at blood glucose levels above 200 mg/dl on at least two consecutive measurements or with blood glucose levels once above 400 mg/dl. All animal experiments were performed as approved by the Landesdirektion Dresden (24-9168.24-1/2014-1, DD 24-5131/338/38 (TVV37/2015).

### DT-mediated Foxp3^+^ Treg Cell Ablation and Adoptive Cell Transfers

Mice were *i.p.* injected with 0.5 μg DT (Merck Millipore - Calbiochem, Darmstadt, Germany) in 200 μl of sterile PBS on two consecutive days, if not stated otherwise. Where indicated, DT-treated NOD.DEREG mice were additionally *i.v.* injected with 5 μg anti-CD3e mAb (145-2C11). For total splenocyte transfers, single-cell suspensions were prepared from pooled spleens of 4-5-week-old (n = 4) and 16-18-week-old (n = 6) NOD.DEREG females, and 5 x 10^6^ total cells were *i.v.* injected into NOD.Rag^–/–^ recipient mice, followed by two consecutive injections of DT at day 7 and 8. For CD4^+^BDC2.5^+^ T cell transfers, conventional T (Tcon) cells (CD4^+^CD62L^hi^Vβ-4^+^CD25^–^GFP^–^) and Foxp3^+^ Treg cells (CD4^+^Vβ-4^+^CD25^high^GFP^+^) were FACS-isolated (99.3 – 99.8% purity) from pooled spleen and LN of NOD.DEREG × BDC2.5 mice. FACS-purified DTR^–^ Treg cells (CD4^+^Vβ-4^+^CD25^high^) from DEREG^–^ NOD.BDC2.5 mice were included to control for DT toxicity. Diabetogenic Tcon cells (5 × 10^5^) were *i.v.* injected into NOD.Rag^–/–^ recipient mice, either alone or co-injected with DTR^+^ or DTR^–^ Treg cells (1 × 10^5^), followed by DT injection on three consecutive days in week 6 after adoptive transfer.

### Immunohistochemistry

Pancreatic cryosections (5μm) were fixed in 4% formalin and stained for C-peptide using polyclonal rabbit anti-C-peptide Ab (Cell Signaling, Germany), followed by Alexa Fluor 488-labeled polyclonal goat anti-rabbit IgG Ab (Invitrogen). Subsequently, detection of CD3 was carried out using rat anti-CD3 mAb (CD3-12) (AbD Serotec), followed by staining with Alexa Fluor 568-labeled polyclonal goat anti-rat secondary Ab (Invitrogen). Nuclei were visualized using 4′-6-Diamidino-2-phenylindole (DAPI). Slides were mounted with Vectashield (Vector Laboratories, Burlingame, CA, USA), using standard protocols. All images were acquired with a Leica SP5 upright Laser Scanning confocal microscope. For evaluation of lymphocyte infiltration (insulitis), at least three sections were collected at 50 μm intervals and 6-12 pictures per pancreas were taken, using the following scale (32): 0, no infiltration; 1, minimal focal infiltration; 2, peri-islet infiltration (<50%); 3, intra-islet infiltration (>50%); 4, extensive infiltration (100%).

### Flow Cytometry and Cell Sorting

Pancreatic islets were isolated by collagenase digestion (0.7 mg/ml) (Sigma-Aldrich Chemie GmbH) and discontinuous Ficoll density gradient. Single-cell suspensions of pancreatic islets and lymphoid tissues were prepared using 70 μm cell strainers (Becton Dickinson, San Diego, CA, USA) and Hank’s buffer [1 x HBSS, 5% (v/v) FCS, 10mM HEPES; all Invitrogen]. Single cell suspensions from spleen were additionally subjected to red blood cell lysis (erythrocyte lysis buffer EL, Qiagen). Peripheral blood mononuclear cells (PBMCs) were obtained by retro-orbital sinus puncture [PBS supplemented with 10% (v/v) Heparin (Biochrom AG, Berlin, Germany)] and Ficoll (VWR, Darmstadt, Germany) gradient centrifugation. mAbs to CD3 (145-2C11), CD4 (RM4-5, GK1.5), CD8 (53–6.7), CD25 (PC61, 7D4), CD44 (IM7), CD62L (MEL-14), Vβ-4 (KT4), and CD49b (R1-2) were purchased from eBioscience (Frankfurt, Germany) or BD (Heidelberg). The samples were analyzed using a LSRII or sorted on a FACS Aria (all BD). Data were analyzed with the FlowJo software (Tree Star).

### Immunophenotyping

For flow cytometry-based immunophenotyping, male and female cohorts of adult, age-matched NOD.DEREG mice were either left untreated or injected with DT on two consecutive days. Pancreatic islets, pancreatic lymph nodes (pLN), and a collection of other lymphoid tissues [subcutaneous LN (scLN), spleen, and thymus] were harvested before (day 0) or at different days after (day 1-7, day 10) administration of the first dose of DT (3-6 mice per timepoint). Single-cell suspensions were subjected to multicolor flow cytometry for the quantification of immune subsets (αβ T cells, NKT cells, and NK cells: mAbs directed against CD3, CD4, CD8, CD25, and CD49b; B cells, granulocytes, macrophages, and dendritic cells: mAbs directed against CD19, Gr1, CD11b, and CD11c).

### Gene Expression Analysis

Freshly isolated pancreata were subjected to rapid freezing and grinding in liquid nitrogen, followed by total RNA extraction using Trizol^®^ (Life Technologies), the RNeasy Mini Kit, and DNase I digestion (Qiagen, Hilden, Germany). Total RNA was extracted from pLN using the RNeasy Mini Kit, DNase I digestion. For real-time RT-PCR, cDNA was synthesized using Oligo-d(T) primers and SuperScript II reverse transcriptase (Invitrogen) according to the manufacturer’s recommendations. The QuantiFast SYBR Green PCR kit (Qiagen) and a Mastercycler ep realplex thermal cycler (Eppendorf) was used to analyze cDNA in replicates. The following primers were used: β-Actin, 5’-TGG AAT CCT GTG GCA TCC ATG AAA C-3’ and 5’- TAA AAC GCA GCT CAG TAA CAG TCC G-3’; GzmA, 5’-TTT CAT CCT GTA ATT GGA CTA A-3’ and 5’-GCG ATC TCC ACA CTT CTC-3’; IFN-*γ*, 5’-GGC TGT TAC TGC CAC GGC ACA-3’ and 5’-CAC CAT CCT TTT GCC AGT TCC TCC-3’; GITR, 5’-GAC GGT CAC TGC AGA CTT TG-3’ and 5’-GCC ATG ACC AGG AAG ATG AC-3’; NKG2D, 5’-ACG TTT CAG CCA GTA TTG TGC-3’ and 5’-GGA AGC TTG GCT CTG GTT C-3’.

## Results

### Generation of Autoimmune Diabetes-Prone DEREG Mice

**Figure 1:**
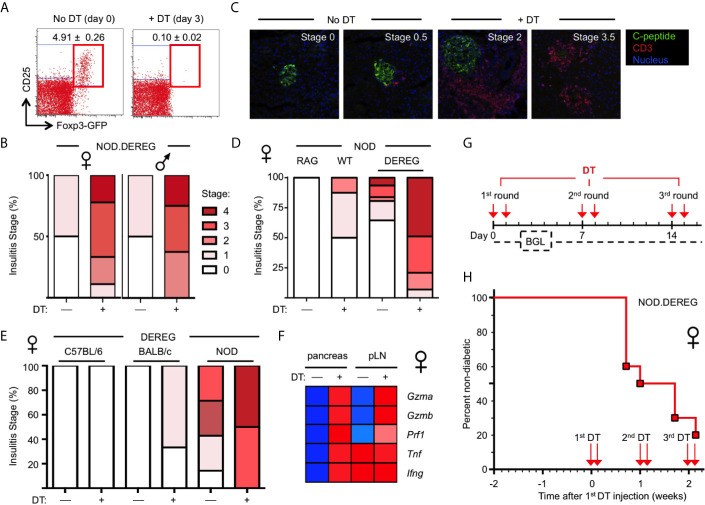
Rapid progression to overt diabetes after acute Treg cell ablation in adult NOD.DEREG females. Mice were either left untreated or injected with DT on two consecutive days, and subjected to further analysis on day 14, unless otherwise stated. **(A)** Efficiency of Treg cell ablation. Representative dot plots of CD4-gated cells from peripheral blood of 16-week-old NOD.DEREG females before (day 0) and after (day 3) injection with DT. Numbers in dots plots indicate mean percentage ± SD of 8 mice (unpaired t-test, p < 0.001) from a single experiment representative of 6 experiments performed (5-9 mice per experiment). **(B–E)** Pancreatic pathohistological changes. Histological sections were scored as described in Materials and Methods. **(B)** Insulitis scores of 4-5-week-old female (left, n = 10) and male (right, n = 9) NOD.DEREG mice. As all mice remained normoglycemic by day 7 after the first DT injection, two additional doses of DT were administered (day 7 and 8) prior to histology on day 14. **(C)** Representative histology and **(D)** insulitis score of 8-9-week-old NOD.DEREG females (8-10 mice per group). Untreated NOD.RAG (n = 4) and DT-injected DEREG^–^ NOD (n = 5) females were included as controls. **(E)** Insulitis scores of 16-week-old DEREG females on different genetic backgrounds (4-7 mice per group). **(F)** Expression of mRNA encoding immune effector molecules with known function in autoimmune β cell destruction. Freshly isolated mRNA from pancreas and pLN (pooled from 4-5 mice per group) of 10-12-week-old NOD.DEREG females was subjected to real-time RT-PCR using β-Actin for normalization. Heat map (row normalized) shows mean values of triplicate samples from a single experiment representative of at least two independent experiments performed. Blue and red represent lowest and highest gene expression values, respectively. Pancreas, Unpaired t-test: p < 0.001: *Gzma, Gzmb, Prf1, Tnf*; p ≤ 0.01: *Ifng*. pLN, Unpaired t-test: p <0.001: *Gzma, Gzmb*; p ≤ 0.01: *Prf1*; not significant: *Tnf, Ifng*. **(G, H)** Manifestation of overt diabetes. **(G)** Cohorts of 16-week-old, initially non-diabetic NOD.DEREG mice were repeatedly injected with 2 doses of DT at 5-day-intervals, as indicated. Blood glucose levels (BGL) were determined at least three times per week. **(H)** Diabetes incidence of female NOD.DEREG mice. Mice were considered diabetic at blood glucose levels above 200 mg/dl on at least two consecutive measurements or with blood glucose levels once above 400 mg/dl. Data are from a single experiment (n = 10) representative of > 8 experiments performed. Note that the diabetes incidence of male NOD.DEREG mice is depicted in **Supplementary Figure S3**.

The DEREG mouse line was originally developed on the non-autoimmune prone C57BL/6 background (9). Here, we introduced the Foxp3^BAC-DTR/GFP^ transgene of DEREG mice into the autoimmune diabetes-susceptible NOD background by extensive backcrossing (see *Materials and Methods*). We preferred this strategy, rather than generating a novel Foxp3-DTR transgenic line directly on the NOD genetic background, because adult DEREG mice on a non-autoimmune prone genetic background have been shown to be resistant to autoimmune diseases or *scurfy*-like symptoms after DT treatment (9, 25, 26). Confirming the validity of our NOD.DEREG model, DT-untreated female mice spontaneously developed overt diabetes, which was accompanied by an early onset of insulitis at ≤ 4 weeks of age and progressive loss of insulin-producing β cells (see below), resulting in the manifestation of hyperglycemia from 12 weeks of age (**Supplementary Figure S1**). In line with previous reports on efficient Treg cell depletion in C57BL/6.DEREG mice (9), administration of two consecutive daily doses of DT into NOD.DEREG mice resulted in an > 98% depletion of CD4^+^GFP^+^ Treg cells in peripheral blood (day 3, **Figure 1A**), while total CD4^+^ T cell proportions (mean ± SD: day 0: 58.3 ± 5.0, day 3: 53.3 ± 4.3, day 7: 52.6 ± 6.0) did not significantly change over time (Unpaired t-test, n = 8). We observed a similar efficiency of DT-mediated Treg cell depletion in both sexes of NOD.DEREG mice, and over a wide age range (4 weeks – 12 months).

### Massive β Cell Loss and Hyperglycemia Within Days after Acute Treg Cell Ablation

In previous studies on a non-autoimmune-prone background, DT-mediated Treg cell depletion resulted in autoimmune diseases only when injected into newborn DEREG mice (9). Consistently, and despite multiple repeated injections of DT, Treg cell depletion in cohorts of young, 4–5-week-old NOD.DEREG mice caused worsening of initially mild insulitis in both sexes (**Figure 1B**), but the majority of mice (≥ 90%) remained normoglycemic and showed no *scurfy*-like symptoms (scaliness and crusting of eyelids/ears/tail, hepatomegaly, splenomegaly, enlarged lymph nodes, early death) (data not shown). In contrast, the pancreas of adult NOD.DEREG females showed strong pathohistological changes, when only two doses of DT were administered on consecutive days, and histological analysis of the pancreas was performed on day 12 (**Figures 1C–E**). This included massive CD3^+^ T cell infiltrates throughout the islet space and complete islet disaggregation with only a few, if any, residual insulin-producing β cells in DT-treated NOD.DEREG mice (**Figure 1C**). In the control cohorts, DT-treated DTR^–^ littermates or sham-injected DTR^+^ NOD mice exhibited no or only minimal peri-islet infiltration (**Figures 1C, D** **)**. With regard to the susceptibility to multiple autoimmune diseases in the NOD background, histological analysis of adult, 9-12-week-old females of our NOD.DEREG colony revealed marked thyroid immune infiltrates, as expected (33), but the severity of autoimmune thyroiditis appeared similar between sham- and DT-injected mice, irrespective of whether they remained nondiabetic or progressed to hyperglycemia (**Supplementary Figure S2A**). We observed only very rare cases (<5%) of autoimmune neuropathy (34) among diabetic Treg cell-depleted mice, as indicated by the manifestation of hind limp paralysis with histological evidence for immune infiltration in the peripheral nerves (**Supplementary Figure S2B**).

Overall, the manifestation of destructive β cell autoimmunity was strictly dependent on the NOD genetic predisposition, as the pancreata of DT-treated DEREG females on the C57BL/6 or BALB/c background were devoid of immune infiltration and evidence for β cell death (**Figure 1E** **).** Notably, DT treatment was found to preserve the age-dependent differences in the severity of spontaneous insulitis that is found in DT-untreated NOD.DEREG females, when comparing different age groups (**Figure 1B**, 4-5 weeks; **Figure 1D**, 8-9 weeks; **Figure 1E**, 16 weeks). Thus, it appears that acute Treg cell ablation in NOD.DEREG mice exacerbates pre-established β cell autoimmunity, while preserving key features of spontaneous diabetes development in the NOD model (35, 36). Consistently, DT treatment of prediabetic NOD.DEREG adults increased mRNA expression of the Th1 cytokines TNF-α and IFN-*γ* selectively in the pancreas, while up-regulation of other autoimmune effector molecules with known functions in β cell destruction could be observed in both pLN and pancreas, such as Granzyme A, Granzyme B or Perforin (**Figure 1F**). The expression of mRNA encoding pro-inflammatory cytokines, such as innate-derived IL-1β or Th2 and Th17 signature cytokines (*e.g.*, IL-4, IL-13, IL-21, IL-22), remained below the detection limit in DT-treated NOD.DEREG mice, but were readily detectable in adolescent Foxp3*^sf^* mice (data not shown). Finally, in cohorts of adult NOD.DEREG mice, ≥ 50% of female (**Figures 1G, H**, *see also* **Figure 3E**) but only ≤ 10% of male (**Supplementary Figure S3**) mice progressed to overt diabetes within 5 days after the administration of two doses of DT. In these experiments, the administration of DT on two consecutive days was required to reproducibly promote overt diabetes at a high incidence. A single dose of DT or the repeated injection of three single doses of DT at 7-day-intervals into cohorts of adult (8-16-week-old) or aged (6-12-months-old) females only sporadically resulted in the induction of diabetes, with an incidence ranging from 0-10% (data not shown).

### Mechanisms That Constrain Autoimmunity in DT-Treated NOD.DEREG Mice

**Figure 2:**
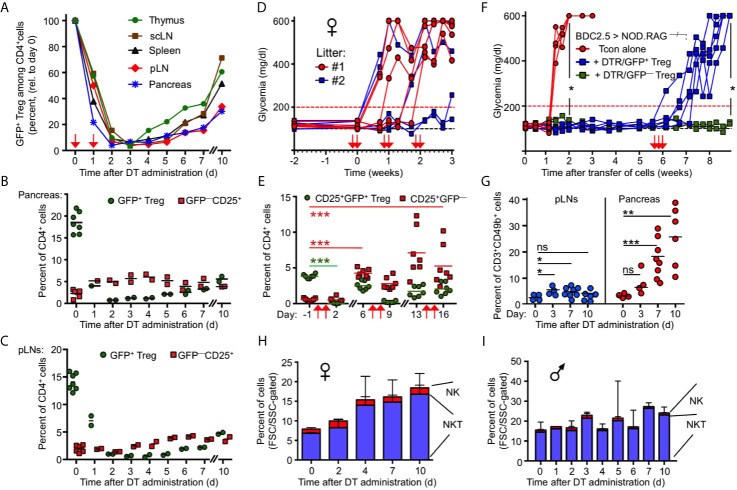
Transient nature of Treg cell ablation in NOD.DEREG mice. **(A)** Kinetics of DT-mediated ablation and subsequent recovery of the CD4^+^GFP^+^ Treg cell compartment in pancreas and indicated lymphoid organs of 10-12-week-old NOD.DEREG mice. Data are from a single experiment (2 mice/timepoint) representative of 3 experiments performed. Arrows indicate days of DT injection. **(B, C)** GFP^+^ Treg cell recovery in **(B)** pancreas and **(C)** pLN is preceded by the rapid accumulation of CD4^+^CD25^+^ cells lacking DTR/GFP expression (GFP^–^). Each symbol corresponds to an individual mouse (10-12-week-old). **(D, E)** Impact of repeated DT administration on glycemic state and Treg cell depletion. **(D)** Blood glucose concentrations of individual NOD.DEREG mice presented in **Figure 1** **(H)**. Arrows indicate days of DT injection. **(E)** Kinetics of CD25^+^GFP^+^ and CD25^+^GFP^–^ cells among CD4-gated cells, as revealed by flow cytometry among peripheral blood-derived CD4^+^ T cells at different timepoints (Two-way ANOVA in combination with Bonferroni’s Multiple Comparison post-test: ***p < 0.001). **(F)** Acute Treg cell ablation in the absence of Treg cell rebound. NOD.RAG1^–/–^ mice were repopulated with diabetogenic BDC2.5 T conventional cells, either alone (red circles) or with GFP^+^ (blue squares) or DTR/GFP^–^ (green squares) Treg cells, and blood glucose levels of recipients were routinely assessed at least twice a week for up to 9 weeks. DT was injected at three consecutive days of week 6 (arrows). (Mann-Whitney-U test; two weeks: p = 0.0159, nine weeks: p = 0.0476). **(G–I)** Pancreatic NKT cell accumulation after Treg cell ablation. **(G)** Percentages of CD3^+^CD49b^+^ NKT cells among FSC/SSC-gated cells in pLNs (left) and pancreas (right) of 12-16-week-old NOD.DEREG females before (day 0) and at indicated days after DT administration. Symbols and horizontal lines indicate individual mice (4-8 mice per timepoint) and mean values, respectively. Unpaired t-test: ns, not significant; *p ≤ 0.05, **p ≤ 0.01, ***p < 0.001). **(H, I)** Kinetics of pancreas-infiltrating CD3^+^CD49b^+^ NKT and CD3^–^CD49b^+^ NK cells (FSC/SSC-gated) in **(H)** female and **(I)** male NOD.DEREG mice (10-12-week-old). Shown are mean percentages ± SD (day 0: n = 7) or mean percentages and range of replicate mice (days 1-7, day 10: n = 2).

In DT-treated NOD.DEREG mice, the manifestation of overt diabetes in the absence of other autoimmune symptoms suggests an intricate balance between diabetogenic and tolerogenic mechanisms, which constrain aggressive autoimmunity primarily to pancreatic β cells. Overall, the efficiency of DT-mediated Treg cell depletion was comparable between different lymphoid tissues and pancreas, reaching its maximum on day 3 after the first of two doses of DT at all anatomical sites (**Figure 2A**). However, the kinetics of Treg cell recovery differed between anatomical sites. Consistent with the thymus as a primary site of Treg cell *de novo* generation, CD4^+^GFP^+^ cells became first detectable in the thymus (day 4, **Figure 2A**), followed by the continuous replenishment of the CD4^+^GFP^+^ Treg cell compartments at peripheral sites, resulting in a recovery rate of 60-70% in spleen and scLN by day 10 (**Figure 2A**). In comparison, the kinetics of GFP^+^ Treg cell recovery in pancreas and pLN was somewhat delayed, reaching ≤ 35% at day 10 (**Figure 2A**), which is likely to facilitate local autoimmune responses, while the rapid GFP^+^ Treg cell recovery in secondary lymphoid tissues is providing a rather narrow time window for the manifestation of systemic autoimmunity.

Given that the endogenous *Foxp3* gene and the Foxp3^BAC-DTR/GFP^ transgene are not physically linked, the activity of Foxp3^+^ Treg cells with a DTR/GFP^–^ phenotype may represent another mechanism that limits catastrophic autoimmunity in DT-treated NOD.DEREG mice. While the expression of GFP and Foxp3 closely correlates in steady-state DEREG mice, DT administration can result in the proliferative expansion of an initially minute population of DT-resistant DTR/GFP^–^Foxp3^+^ Treg cells (37). In fact, 43.1 ± 3.5% (mean ± SD, n = 3) of pancreatic CD4^+^CD25^+^GFP^–^ cells expressed Foxp3 protein at day 5 after DT administration, as compared to 9.1 ± 1.5% (mean and range of duplicate samples) in DT-untreated NOD.DEREG mice, as judged by the analysis of Foxp3 protein expression using anti-Foxp3 mAb (data not shown). Furthermore, the restoration of a pancreatic GFP^+^ Treg cell compartment was preceded by the accumulation of such DTR/GFP^–^CD25^+^ T cells within 1-2 days after DT administration (**Figure 2B**). DT-resistant DTR/GFP^–^CD25^+^ T cells also accumulated at other anatomical sites of DT-treated NOD.DEREG mice, although with delayed kinetics (**Figure 2C**). The rapid dynamics of Treg cell rebound was further illustrated by longitudinal studies concerned with the impact of prolonged DT administration (**Figures 1H**) on the progression to hyperglycemia (**Figures 2D**) and the GFP^–^/GFP^+^ Treg cell compartment size in peripheral blood (**Figure 2E**) of individual mice. Consistent with our data on pancreas (**Figure 2B**) and lymphoid tissues (**Figures 2A, C** **)**, the population size of both GFP^–^ and GFP^+^ cells in blood markedly increased by day 6 after the first round of two DT doses (**Figure 2E**). The repeated injection of two doses of DT at 5-day intervals further enhanced the accumulation of DT-resistant CD25^+^DTR/GFP^–^ Treg cells, while the depletion efficiency of the CD25^+^DTR/GFP^+^ compartment appeared to decrease (**Figure 2E**).

Next, we aimed to assess the impact of DT-mediated Treg cell depletion on β cell autoimmunity mediated by defined numbers of diabetogenic T cells, in the absence of potentially confounding effects of ‘rebounding’ Treg cells. For this, immunodeficient NOD.Rag1^–/–^ mice (no B and T/NKT cells) were reconstituted with 5 x 10^5^ diabetogenic CD4^+^BDC2.5^+^ T cells, either alone or with small numbers of DTR/GFP^+^ Treg cells (5 x 10^4^ cells), followed by the injection of DT in week 6 (**Figures 2F**). In this experimental setting, the *de novo* generation of Foxp3^+^ Treg cells is precluded by the complete block of thymic T cell development in NOD.Rag1^–/–^ recipients, and DT-resistant DTR/GFP^–^ Treg cells were excluded by FACS-based isolation of GFP^+^ Treg cells prior to adoptive transfer. While co-transferred Treg cells efficiently interfered with the manifestation of overt diabetes, all recipients of DTR/GFP^+^ Treg cells nearly synchronously developed severe hyperglycemia within 1 week after DT-mediated depletion (**Figure 2F**). DT-treated NOD.Rag1^–/–^ recipients of diabetogenic CD4^+^BDC2.5^+^ T cells and DTR^–^ Treg cells remained normoglycemic, excluding a major role of DT toxicity on β cell death (**Figure 2F**).

Overall, these data highlight the continuous requirement for Treg cell-mediated suppression in the control of destructive β cell autoimmunity, and suggest Treg cell rebound as plausible mechanism underlying the incomplete diabetes penetrance in DT-treated NOD.DEREG mice (**Figure 1H**). Additionally, kinetics studies employing flow cytometric immunophenotyping (see Materials and Methods) indicated that CD3^+^CD49b^+^ NKT cells were initially less abundant in the pancreas of female NOD.DEREG mice, but their population size gradually increased after DT administration (**Figures 2G, H** **)**, whereas the pancreatic NKT cell compartment in males remained largely constant (**Figure 2I**). Although their exact role in T1D has been controversially discussed (38–41), these data suggest that NKT cells may exert a tolerogenic function in Treg cell-depleted NOD.DEREG females.

### Anti-CD3 mAb Therapy Following Treg Cell Depletion Interferes With Diabetes

**Figure 3:**
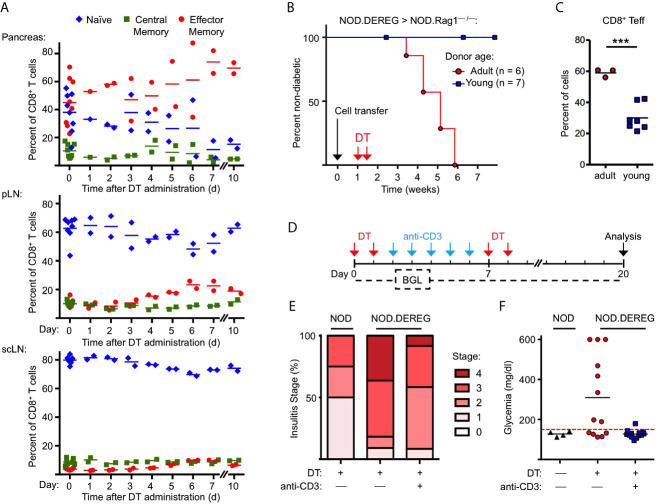
Anti-CD3 mAb therapy after Treg cell ablation ameliorates insulitis and prevents progression to hyperglycemia. **(A)** Kinetics of CD8^+^ T cells with a naïve (CD62L^high^CD44^low^), central memory (CD62L^int^CD44^int^), and effector/memory (CD62L^low^CD44^high^) phenotype in pancreas (top), pLN (middle), and scLN (bottom) of 10-12-week-old NOD.DEREG mice before (day 0) and at indicated days after DT administration. **(B, C)** Adoptive splenocyte transfer model. **(B)** Splenocytes from adult (16-18-week-old) but not young (4-5-week-old) DTR/GFP^+^ NOD.DEREG donors promote overt diabetes in NOD.RAG1^–/–^ recipient mice after DT treatment. Black arrow indicates the day of splenocyte transfer (2 x 10^6^ total cells), and red arrows indicate the days of DT administration. (Log-Rank (Mantel-Cox) test, p = 0.0097). **(C)** Proportions of CD8^+^ T cells with a CD62L^low^CD44^high^ effector/memory phenotype in the pLN of NOD.RAG1^–/–^ recipients of splenocytes from young (red circles) or adult (blue squares) NOD.DEREG donors. Unpaired t-test: ***p < 0.001. **(D–F)** anti-CD3 mAb treatment of Treg cell-depleted NOD.DEREG mice. **(D)** Scheme of experimental design. Cohorts of adult NOD.DEREG females were injected with DT only (n = 13), or were additionally treated with anti-CD3 mAb (n = 13). Untreated DTR/GFP^–^ NOD.DEREG mice were included for comparison (n = 4). Arrows: red, DT injection; blue, anti-CD3 injection; black, analysis by histology and flow cytometry. Blood glucose concentrations were assessed before (day 0) and every second day after the first injection with DT until the end of the observation period on day 14. **(E)** Histological insulitis score of indicated experimental groups, and **(F)** blood glucose levels of individual mice (one-way ANOVA: p ≤ 0.01). Each symbol in **(A, C, F)** corresponds to an individual mouse.

With the exception of NKT cells (*see* **Figure 2G, H**), our flow cytometric immunophenotyping revealed no other quantitative changes of major immune cell subsets in the pancreas of NOD.DEREG mice (data not shown). This also holds true for CD3^–^CD49b^+^ NK cells (**Figure 2H**), which have previously been shown to undergo massive proliferative expansion (up to 5-fold) within 48 hours after DT-mediated Treg cell depletion in a Foxp3^BAC-DTR/GFP^ NOD mouse line carrying the diabetogenic BDC2.5 TCR as additional transgene (30). Additionally, acute Treg cell ablation in adult NOD.DEREG mice had no impact on the population size of pancreatic CD4^+^ and CD8^+^ T effector cells (**Supplementary Figure S4**), which are thought to play a major role as physiologic mediators of β cell destruction in the NOD model (2, 35). However, DT administration increased the frequency of CD8^+^ T cells with an effector/memory phenotype, in pancreas (**Figure 3A**) and pLN (**Figure 3B**), and to a lesser extend in scLN (**Figure 3C**), which could be attributed to an increase in the compartment size of CD62L^low^CD44^high^ effector/memory T cells at the expense of CD62L^high^CD44^low^ naïve T cells. We made similar observations for conventional CD4^+^ T effector cells (**Supplementary Figure S5**), but anti-CD4 mAb (GK1.5) administration into cohorts of Treg cell-depleted NOD.DEREG mice (n = 10) did not appreciably interfere with the manifestation of overt diabetes, whereas anti-CD8α mAb (53.6.72) administration reduced the diabetes incidence to 10%, as compared to 50% in untreated and 60% in anti-CD4 mAb-treated mice (data not shown). One interpretation of these data is that CD4^+^ T cells are dispensable at this stage of the disease, while CD8^+^ T cells represent the final effector cells. As the CD8α chain is not exclusive to αβ T cells, but also expressed on other immune cells (such as NKT or DCs), we next assessed the diabetogenic potential of NOD.DEREG splenocytes after injection into lymphopenic NOD.Rag1^–/–^ mice (**Figure 3D**). In this adoptive transfer model, the manifestation of overt diabetes has been shown to be strictly CD8^+^ T cell-depend, with numbers of diabetogenic CD8^+^ T cells closely correlating with the kinetics of β cell destruction (42). Our results show that DT administration into NOD.Rag1^–/–^ recipients of splenocytes from cohorts of adult NOD.DEREG females promotes overt diabetes in all recipients (**Figure 3B**), which correlated with an enrichment of effector/memory-type CD8^+^ T cells in the pLN (**Figure 3C**) but not scLN (data not shown). In contrast, all NOD.Rag1^–/–^ recipient mice of splenocytes from young NOD.DEREG donors maintained normoglycemia during the entire observation period of 8 weeks (**Figure 3B**). These data further indicate that CD8^+^ T cells are key mediators of destructive β cell autoimmunity in the NOD.DEREG model, and provide a mechanistic basis for our observation that young, DT-treated NOD.DEREG mice are largely refractory to the manifestation of diabetes (**Figure 1B**).

We also assessed the impact of anti-CD3 mAb therapy on β cell autoimmunity in Treg cell-depleted NOD.DEREG mice (**Figure 3D**). In the NOD model, treatment with anti-CD3 mAb at recent diabetes onset has been shown to restore normoglycemia and long-term immune tolerance, but was ineffective in preventing destructive β cell autoimmunity, when injected at earlier stages of diabetes development (43, 44). Our results show that anti-CD3 mAb administration following DT administration ameliorated the strong pathohistological changes observed in the pancreas of Treg cell-depleted cohorts of adult NOD.DEREG females (**Figure 3E**) and interfered with the manifestation of hyperglycemia in all anti-CD3-treated mice (**Figure 3F**). Normoglycemia was also maintained when DT was repeatedly injected after discontinuation of anti-CD3 mAb treatment (**Figures 3D, F** **)**, indicating that anti-CD3 mAb-mediated β cell protection is independent of repopulating GFP^+^ Treg cells.

## Discussion

Here, we show that the specific ablation of Foxp3^+^ Treg cells in DEREG mice can reproducibly precipitate severe insulitis and stable hyperglycemia in the context of a polyclonal TCR repertoire, provided that the autoimmune susceptibility is pre-installed by the NOD genetic background. One of the strengths of the NOD.DEREG model is that it preserves key aspects of the physiologic disease course in Treg cell-proficient NOD mice (*e.g.*, Th1 bias, role of CD8^+^ T cells, female sex bias), while the transient nature of DT-mediated Treg cell depletion minimizes potentially confounding effects of systemic autoimmunity. The reappearance of Foxp3^+^ Treg cells shortly after withdrawal of DT has also been observed in other Foxp3^DTR^ lines (8, 45), including Foxp3^BAC-DTR/GFP^ mice (9), but the apparent differences in the depletion efficiency and recovery kinetics between mouse lines indicate that the DEREG model is particularly suitable for studies on the Treg cell-mediated control of organ-specific autoimmune responses (26).

Overall, our findings indicate that the diabetes incidence in Treg cell-depleted NOD.DEREG mice is largely determined by the extent of preformed pancreatic lesions and numbers of diabetogenic CD8^+^ T cells at the time of DT administration. This is in line with the observation that Foxp3^*sf*^ mice (11) and 4-5-week-old, DT-treated NOD.DEREG mice (**Figure 1B**) are refractory to the manifestation of severe insulitis, despite a comparable efficiency in the DT-mediated depletion of the pancreatic Treg cell compartment in young and adult NOD.DEREG mice (data not shown). However, the severity of destructive β cell autoimmunity in DT-treated adult NOD.DEREG females appears rather unexpected, in particular in light of previous studies on Foxp3^+^ Treg cell targeting using anti-CD25 mAb and the moderate spontaneous diabetes incidence that we observed in the present study in DT-untreated NOD.DEREG mice (**Supplementary Figure S1A**). Importantly, and consistent with strong pancreatic lesions (**Figures 1C–E**), many Treg cell-depleted NOD.DEREG mice nearly synchronously progressed to overt diabetes within 3 days after the administration of only 2 doses of DT (**Figure 2D**). In fact, this rapid kinetics was comparable to previous observations made in BDC2.5 TCR-transgenic DEREG (31)and NOD^BAC-DTR/GFP^ mice (30). We also addressed the possibility that alleviating the metabolic stress of residual β cells by insulin replacement therapy for the duration of Treg cell recovery may help restoring metabolic homeostasis (*e.g.*, by promoting the regeneration of functional β cells) (46, 47). However, the administration of exogenous insulin into DT-treated NOD.DEREG mice shortly after diagnosis of overt diabetes restored normoglycemia for several weeks, but all mice returned to high blood glucose levels, once insulin therapy was discontinued (data not shown), further illustrating the destructiveness of β cell autoimmunity.

Our data suggest a scenario, in which pancreatic Foxp3^+^ Treg cells in prediabetic NOD.DEREG mice interfere rather late in the cascade of events ultimately leading to the spontaneous progression of overt diabetes, highlighting the important role of continuous Treg cell activity in constraining terminal β cell destruction by pancreas-infiltrating CD8^+^ T cells. This interpretation is further supported by our observation that, in contrast to anti-CD4 mAb, anti-CD8 and anti-CD3 mAb (44) administration into DT-treated NOD.DEREG mice interfered with the progression to overt diabetes (**Figure 3F**), despite substantial histopathological changes (**Figure 3E**). In this context, anti-CD3 mAb is of particular interest, as targeting CD3 is a promising approach currently being pursued for the therapy of human T1D (48, 49). Several non-mutually exclusive mechanisms underlying the action of anti-CD3 mAb therapy have been proposed, including the induction of recessive tolerance in pathogenic T effector cells (50), and of dominant tolerance by promoting Foxp3^+^ Treg cell activity (51, 52). Notably, in the BDC2.5-transgenic NOD*^Rag^* model of autoimmune diabetes, anti-CD3 treatment has been shown to induce massive proliferation of an initially constrained population of BDC2.5^+^Foxp3^+^ Treg cells and long-term protection from diabetes development, which could be abrogated by subsequent DT-mediated Treg cell depletion (51). Here, we show that anti-CD3 mAb therapy in Treg cell-depleted NOD.DEREG mice potently interfered with diabetes development (**Figures 3E, F**), probably by mechanisms independent of Foxp3^+^ Treg cells. One plausible explanation of these data is that anti-CD3 mAb can also exert its protective effect by acting on diabetogenic CD8^+^ T cells (49, 53, 54). Clearly, further studies are warranted to more precisely determine the relative contribution of recessive and dominant tolerance mechanisms to the anti-CD3 mAb-mediated effects on β cell autoimmunity.

In summary, the NOD.DEREG line represents a novel tool to analyze the specific role of Foxp3^+^ Treg cells in the control of β cell autoimmunity, resolving some of the previous limitations of NOD mice with constitutive Foxp3 deficiency or transgenic expression of a diabetogenic TCR. This includes mechanistic studies on novel Treg cell-based therapies under experimental conditions of synchronized, advanced β cell autoimmunity.

## Data Availability Statement

The original contributions presented in the study are included in the article/**Supplementary Material**. Further inquiries can be directed to the corresponding author.

## Ethics Statement

The animal study was reviewed and approved by Landesdirektion Dresden, Germany.

## Author Contributions

DW designed, performed, and analyzed the experiments, and contributed to the data interpretation and assisted in manuscript preparation. MJan, MJay, FP, MW, CP, AH contributed to the acquisition, analysis, and interpretation of data. TS, EB, and KK conceived the research. KK guided its design, analysis and interpretation, and wrote the manuscript. All authors contributed to the article and approved the submitted version.

## Funding

This work was supported by the FZT 111 (CRTD/Center for Regenerative Therapies Dresden, DFG), and by a BMBF (German Ministry for Education and Research) grant to the German Center for Diabetes Research (DZD e.V., FKZ01GI0924).

## Conflict of Interest

The authors declare that the research was conducted in the absence of any commercial or financial relationships that could be construed as a potential conflict of interest.

## Publisher’s Note

All claims expressed in this article are solely those of the authors and do not necessarily represent those of their affiliated organizations, or those of the publisher, the editors and the reviewers. Any product that may be evaluated in this article, or claim that may be made by its manufacturer, is not guaranteed or endorsed by the publisher.
