# Supplementary figures and images for: Transient Depletion of Foxp3+ Regulatory T Cells Selectively Promotes Aggressive β Cell Autoimmunity in Genetically Susceptible DEREG Mice
Source: Front Immunol. 2021 Aug 10;12:720133. doi: 10.3389/fimmu.2021.720133 (PMC8382961; doi:10.3389/fimmu.2021.720133)

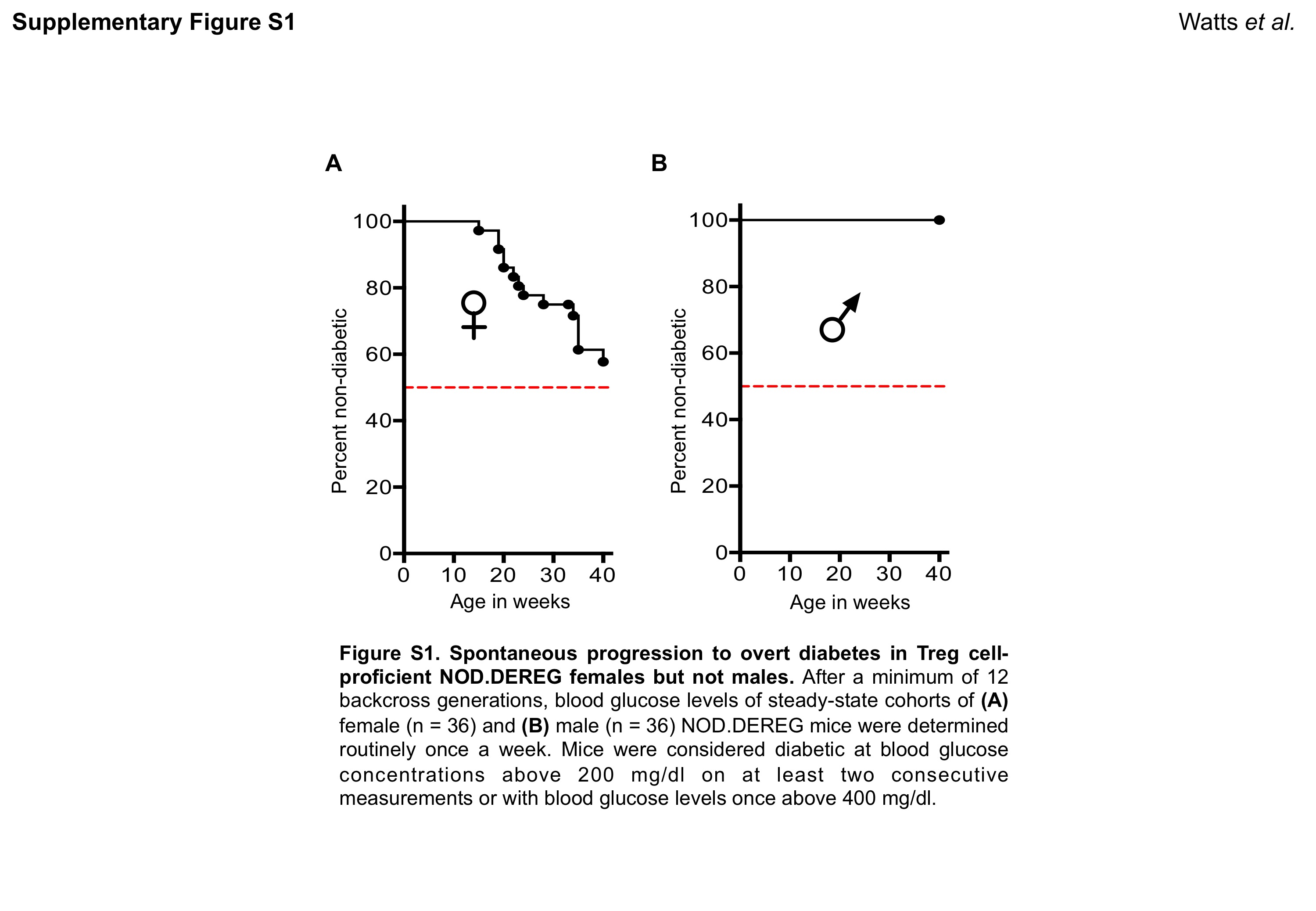

Supplement: Supplementary file 1 [file Image_1.jpeg]

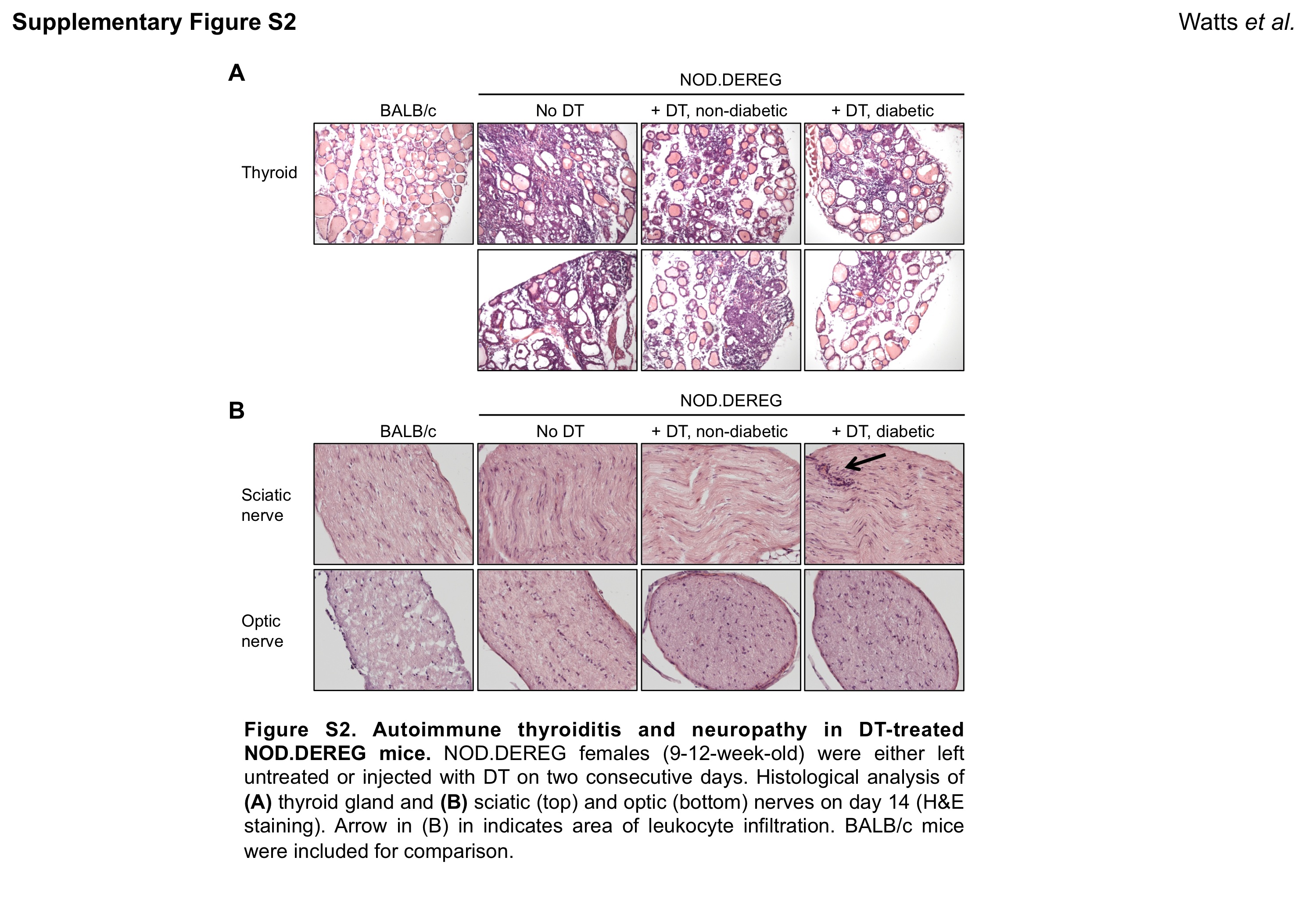

Supplement: Supplementary file 2 [file Image_2.jpeg]

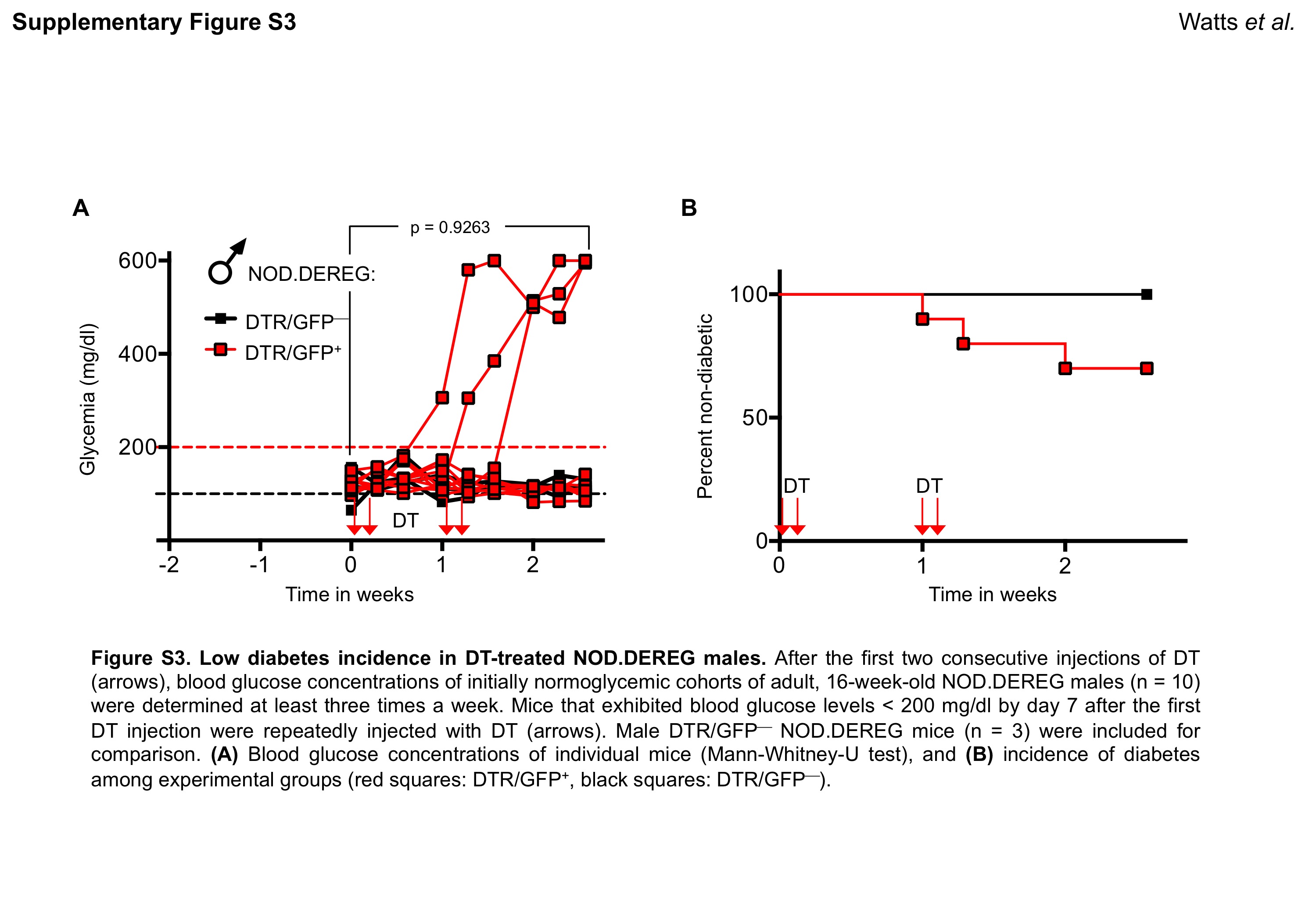

Supplement: Supplementary file 3 [file Image_3.jpg]

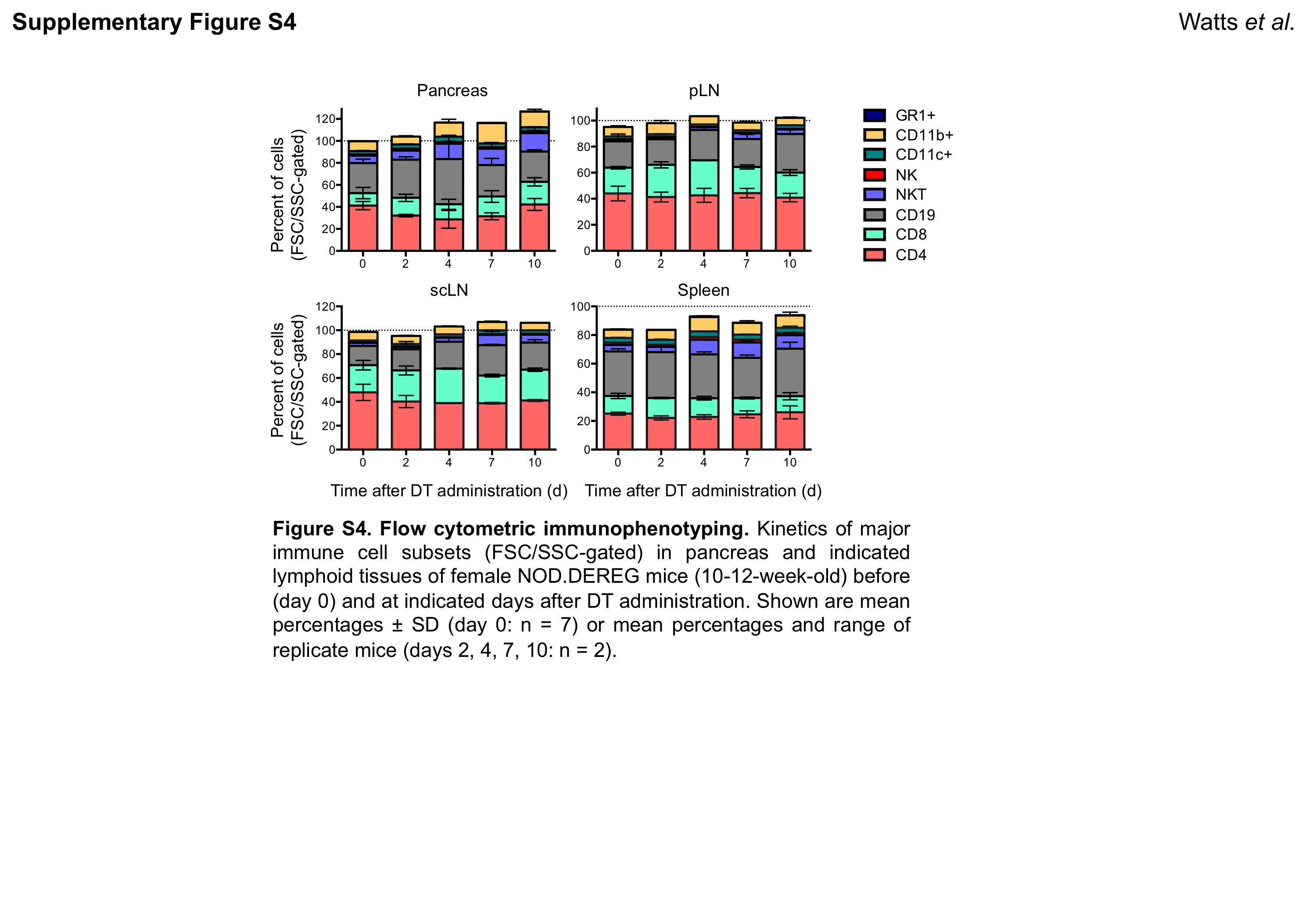

Supplement: Supplementary file 4 [file Image_4.jpeg]

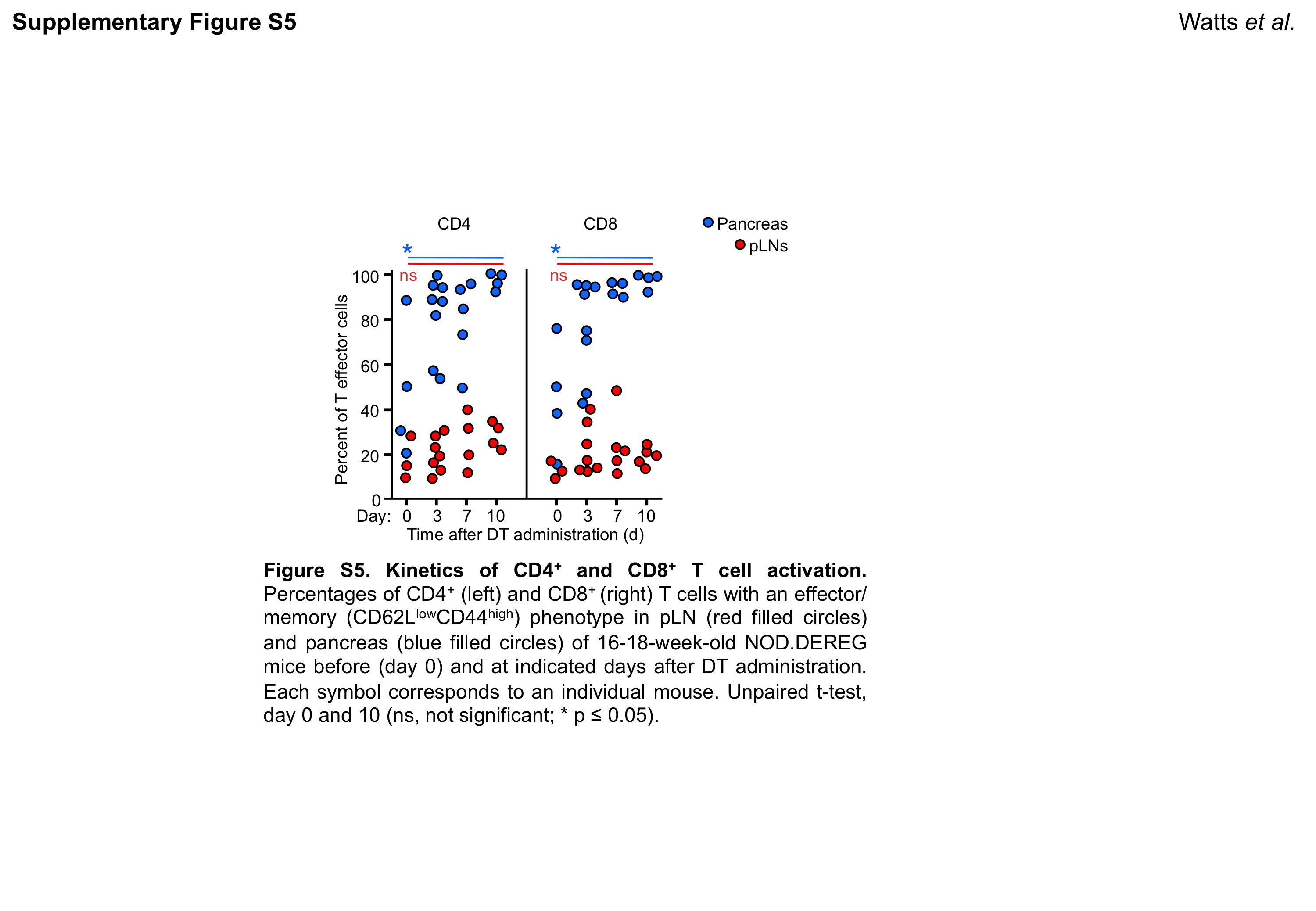

Supplement: Supplementary file 5 [file Image_5.jpeg]
